# Effect of cytomegalovirus reactivation on the time course of systemic host response biomarkers in previously immunocompetent critically ill patients with sepsis: a matched cohort study

**DOI:** 10.1186/s13054-018-2261-0

**Published:** 2018-12-18

**Authors:** Kirsten van de Groep, Stefan Nierkens, Olaf L. Cremer, Linda M. Peelen, Peter M. C. Klein Klouwenberg, Marcus J. Schultz, C. Erik Hack, Tom van der Poll, Marc J. M. Bonten, David S. Y. Ong, on behalf of the MARS consortium, Friso M. de Beer, Lieuwe D. J. Bos, Gerie J. Glas, Arie J. Hoogendijk, Roosmarijn T. M. van Hooijdonk, Janneke Horn, Mischa A. Huson, Nicole P. Juffermans, Tom van der Poll, Laura R. A. Schouten, Brendon Scicluna, Marcus J. Schultz, Marleen Straat, Lonneke A. van Vught, Luuk Wieske, Maryse A. Wiewel, Esther Witteveen, Marc J. M. Bonten, Olaf L. Cremer, Jos F. Frencken, K. van de Groep, Peter M. C. Klein Klouwenberg, Maria E. Koster-Brouwer, David S. Y. Ong, Meri R. J. Varkila, Diana M. Verboom

**Affiliations:** 1 Department of Epidemiology, Julius Center for Health Sciences and Primary Care, University Medical Center Utrecht, Utrecht University, P.O. Box 85500, 3508 GA Utrecht, the Netherlands; 2 Department of Intensive Care Medicine, University Medical Center Utrecht, Utrecht University, Heidelberglaan 100, 3584 CX Utrecht, the Netherlands; 3 Laboratory of Translational Immunology, University Medical Center Utrecht, Utrecht University, Heidelberglaan 100, 3584 CX Utrecht, the Netherlands; 4 Department of Intensive Care, Amsterdam University Medical Centers, University of Amsterdam, Meibergdreef 9, 1105 AZ Amsterdam, the Netherlands; 5 Center of Experimental and Molecular Medicine, Amsterdam University Medical Centers, University of Amsterdam, Meibergdreef 9, 1105 AZ Amsterdam, the Netherlands; 6 Division of Infectious Diseases, Amsterdam University Medical Centers, University of Amsterdam, Meibergdreef 9, 1105 AZ Amsterdam, the Netherlands; 7 Division of Medical Microbiology, University Medical Center Utrecht, Utrecht University, Heidelberglaan 100, 3584 CX Utrecht, the Netherlands; 8 Department of Medical Microbiology and Infection Control, Franciscus Gasthuis & Vlietland, Kleiweg 500, 3045 PM Rotterdam, the Netherlands

**Keywords:** Cytomegalovirus, Reactivation, Host response, Inflammation, Critically ill

## Abstract

**Background:**

Cytomegalovirus (CMV) reactivation in previously immunocompetent critically ill patients is associated with increased mortality, which has been hypothesized to result from virus-induced immunomodulation. Therefore, we studied the effects of CMV reactivation on the temporal course of host response biomarkers in patients with sepsis.

**Methods:**

In this matched cohort study, each sepsis patient developing CMV reactivation between day 3 and 17 (CMV+) was compared with one CMV seropositive patient without reactivation (CMVs+) and one CMV seronegative patient (CMVs−). CMV serostatus and plasma loads were determined by enzyme-linked immunoassays and real-time polymerase chain reaction, respectively. Systemic interleukin-6 (IL-6), IL-8, IL-18, interferon-gamma–induced protein-10 (IP-10), neutrophilic elastase, IL-1 receptor antagonist (RA), and IL-10 were measured at five time points by multiplex immunoassay. The effects of CMV reactivation on sequential concentrations of these biomarkers were assessed in multivariable mixed models.

**Results:**

Among 64 CMV+ patients, 45 could be matched to CMVs+ or CMVs− controls or both. The two baseline characteristics and host response biomarker levels at viremia onset were similar between groups. CMV+ patients had increased IP-10 on day 7 after viremia onset (symmetric percentage difference +44% versus −15% when compared with CMVs+ and +37% versus +4% when compared with CMVs−) and decreased IL-1RA (−41% versus 0% and −49% versus +10%, respectively). However, multivariable analyses did not show an independent association between CMV reactivation and time trends of IL-6, IP-10, IL-10, or IL-1RA.

**Conclusion:**

CMV reactivation was not independently associated with changes in the temporal trends of host response biomarkers in comparison with non-reactivating patients. Therefore, these markers should not be used as surrogate clinical endpoints for interventional studies evaluating anti-CMV therapy.

**Electronic supplementary material:**

The online version of this article (10.1186/s13054-018-2261-0) contains supplementary material, which is available to authorized users.

## Introduction

Cytomegalovirus (CMV) reactivation is observed in 14–41% of intensive care unit (ICU) patients without known prior immune deficiency [1–3] and is associated with increased morbidity and mortality [4–6]. In a previous study, we estimated that the population-attributable fraction of ICU mortality due to CMV reactivation was 23% in patients with acute respiratory distress syndrome (ARDS) [7]. In a subsequent study among patients with septic shock, we found an effect of CMV reactivation on ICU mortality only in patients with concurrent Epstein–Barr virus reactivation [8]. Although multiple studies point toward a causal relationship, definitive proof that CMV reactivation worsens clinical outcome is lacking, as most data are also compatible with a scenario in which CMV reactivation is merely a marker of immune suppression in this patient group.

Based on previous studies in ICU patients, there is a clear pathophysiological link between inflammation and immune suppression on the one hand and the subsequent risk of CMV reactivation on the other [9–13]. Markers reflecting impaired functioning of natural killer cells and cytotoxic T cells were predictive of CMV reactivation [10, 11]. Furthermore, bacterial sepsis and corticosteroids have been identified as clinical risk factors for CMV reactivation [9, 12, 13]. However, less is known about the reverse association and thus the effects of CMV reactivation on the immune system. Direct cytotoxic effects of CMV on organs have been observed primarily in immunocompromised hosts [14] but also in previously immunocompetent patients in the ICU [15]. Moreover, indirect immune-modulating effects are assumed to play a role in the pathogenicity of CMV [13, 16–18].

*In vitro* analysis revealed multiple mechanisms encoded within the genome of CMV that may contribute to a non-specific inhibition of both cellular and humoral immunity [19]. Observational clinical studies yielded conflicting results comparing levels of multiple inflammatory markers in patients with and without CMV reactivation [1, 11, 20]. However, these studies analyzed biomarker responses only immediately upon ICU admission and thus could not assess potential immunological effects due to the onset of CMV reactivation. Nevertheless, cytokine levels were used as a primary (surrogate) endpoint in a recent placebo-controlled randomized control trial in which prophylactic antiviral treatment with ganciclovir failed to reduce interleukin-6 (IL-6) levels [21]. Hence, definite proof of immune-modulating effects induced by CMV remains to be demonstrated. Naturally, such an effect can be demonstrated only after onset of CMV reactivation. Therefore, this longitudinal study aimed to investigate whether the temporal course of seven host response biomarkers, including both pro- and anti-inflammatory cytokines, in previously immunocompetent ICU patients with sepsis differs between patients with and without CMV reactivation.

## Methods

### Study population

This matched cohort study was performed among patients who had been included in two previous studies conducted within the Molecular Diagnosis and Risk Stratification of Sepsis (MARS) cohort [7, 8]. For this study, we included sepsis patients who presented with either concomitant ARDS or septic shock to the mixed ICUs of two university medical centers in the Netherlands between January 2011 and June 2014 and had remained in the ICU beyond day 4. Exclusion criteria were CMV seronegative patients with CMV viremia (thus a primary infection) during their ICU stay and known immunodeficiency or anti-viral treatment in the week before ICU admission. The institutional review boards of both study centers approved an opt-out method of informed consent (protocol number 10-056C).

From this parent cohort, we selected patients with an onset of CMV reactivation between day 3 and 17 in the ICU. These patients with viremia were matched to two control groups consisting of patients without viremia on any day of ICU admission. First, we matched patients with reactivation in a 1:1 ratio to CMV seropositive patients without reactivation (further referred to as “primary comparison”). Second, we matched patients with reactivation in a 1:1 ratio to CMV seronegative patients without CMV viremia (further referred to as “secondary comparison”). This secondary comparison was intended mainly to confirm results of the primary comparison; the rationale was that any finding suggestive for an effect of CMV reactivation should also become apparent when compared with seronegative patients who are not at risk for CMV reactivation. Matching criteria to reduce confounding were length of stay until reactivation (determines *t* = 0), Sequential Organ Failure Assessment (SOFA) score at *t* = 0 (± 2 points), age (± 10 years), sex, and high-dose corticosteroid use during 4 days prior to *t* = 0 (that is, more than 250 mg hydrocortisone or equivalent). Patients were also matched on hospital and calendar day of ICU admission (± 365 days) in order to reduce possible influences of variation in sample workup and biobank storage duration [22]. The optimal matching result was retrieved by selecting the largest sample size after 1000 random iterations of the matching procedure.

### Measurements

Leftover plasma, obtained daily as part of routine patient care, was stored at −80 °C and used to determine CMV serostatus at ICU admission. Subsequently, CMV load in blood was measured weekly, and for intermediary days, on which quantitative polymerase chain reaction was not performed, we estimated viral loads by log-linear imputation (see electronic supplementary materials of [7]). CMV viremia was defined as at least 100 international units (IU) per milliliter. This cutoff value was similar to the ones used in previous studies [7, 8]. Results of CMV viral load measurements in plasma performed for this study were not made available to the treating physicians, and none of the included patients received anti-CMV treatment. To map the immune response, we measured a panel of host response biomarkers in samples derived from five time points: day of viremia onset (*t* = 0), 2 days prior (*t* = −2), and after viremia onset at day 3, 7, and 10 (sample availability depended on length of stay in the ICU). A multiplex luminex immunoassay was performed by using EDTA plasma and included the following proteins: IL-6, IL-8, IL-18, tumor necrosis factor-alpha (TNF-α), TNF-related apoptosis-inducing ligand (TRAIL), interferon-gamma (IFN-γ), IFN-γ–induced protein-10 (IP-10), neutrophilic elastase, granzyme-B, IL-1 receptor antagonist (RA), and IL-10. Based on the results of a pilot run using 82 samples obtained from 15 ARDS patients without sepsis at ICU admission (whom were not included in this study), we excluded IFN-γ, TNF-α, TRAIL, and granzyme-B from the final panel because the levels of these biomarkers were below the lower limit of detection in more than 70% of the samples. Of note, in this pilot run, CMV reactivation was not associated with detectability of the four excluded biomarkers.

Measurements of biomarkers were performed by using an in-house developed and validated multiplex immunoassay (ISO9001 certified) based on Luminex technology (xMAP, Luminex, Austin, TX, USA). The assay was performed as described previously [23]. In short, thawed EDTA plasma samples (60 μL) were diluted 1:1 in High Performance Elisa (HPE) buffer (Sanquin, the Netherlands) and centrifuged through filtration columns to remove debris. Then non-specific heterophilic immunoglobulins were pre-absorbed from all samples with HeteroBlock (Omega Biologicals, Bozeman, MT, USA). Next, samples were incubated with antibody-conjugated MagPlex microspheres for 1-h at room temperature with continuous shaking and this was followed by 1-h incubation with biotinylated antibodies and 10-min incubation with phycoerythrin-conjugated streptavidin diluted in HPE buffer. Acquisition was performed with the FLEXMAP 3D system (Bio-Rad Laboratories, Hercules, CA, USA) in combination with xPONENT software version 4.2 (Luminex). Data were analyzed by 5-parametric curve fitting using Bio-Plex Manager software, version 6.1.1 (Bio-Rad Laboratories).

### Statistical analysis

Univariable analyses were performed to compare patients and disease characteristics for matched groups with and without CMV reactivation using chi-squared, Wilcoxon rank sum, or Fischer exact tests as appropriate. Measured host response markers were natural log-transformed concentrations in picograms per milliliter for all analyses. Symmetric percentage differences were calculated for each patient at the different time points. This delta percentage reflects the relative change from the measurement 2 days prior to CMV reactivation until the follow-up measurement [24].

We performed additional multivariable analyses by using generalized linear mixed models to assess the effect of CMV reactivation on the time course of each individual biomarker. In the mixed model analyses, we assessed whether baseline biomarker levels were comparable between matched groups (that is, coefficient for CMV reactivation) as well as the effect of CMV on the course of the biomarker levels over time (that is, coefficient for interaction term between time and CMV reactivation). *A priori* we chose to model the established immune markers IL-6 and IL-10. Based on the observed divergence in the symmetric percentage differences over time between groups, we conducted the multivariable analyses also for the pro-inflammatory chemokine IP-10 and the anti-inflammatory cytokine IL-1RA. Since not all CMV reactivation patients were included in both comparisons, we performed separate mixed model analyses for the primary and secondary comparisons. Thus, in total, eight models were built (for each of the four biomarkers in each of the two comparisons). For each model, SOFA score at *t* = 0 (that is, the day of reactivation) and age were included as confounders since we used a range (instead of an exact value) as matching criteria for these co-variables. For the fixed part of th emodels a polynomial term for time was evaluted (that is, quadratic time effect). Furthermore, a random intercept and a rondom slowe were evaluted for each model. Restricted maximum likelihood estimation (REML) was used to generate unbiased variance estimates for the final models [25]. Different ways to model the time course for each host response marker were compared by using the likelihood ratio test and Akaike’s information criterion.

To take multiple testing into account and reduce the risk of spurious findings, we performed all statistical testing against a *P* value of 0.01 and used a confidence interval of 99%. Bonferroni adjustment was deemed inappropriate and too conservative as the different measurements performed over time within a single patient and hence the tests were highly correlated with each other. Analyses were performed by using either SAS Enterprise Guide 7.1 (SAS Institute, Cary, NC, USA) or R version 3.3.2 (R Foundation for Statistical Computing, 2015; used packages “lme4”, “lmetest”). Figures were made using GraphPad Prism version 7.04 (GraphPad Software, La Jolla, CA, USA).

## Results

**Fig. 1:**
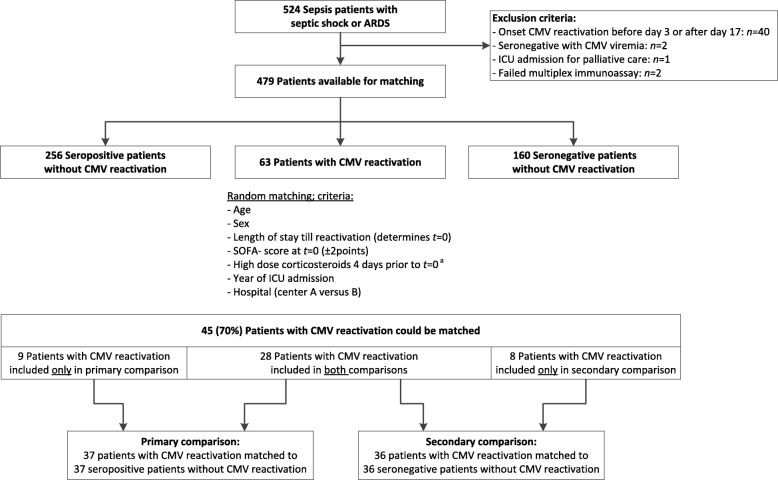
Patient inclusion. Abbreviations: *ARDS* acute respiratory distress syndrome, *CMV* cytomegalovirus, *ICU* intensive care unit, *SOFA* sequential organ failure assessment. ^a^High-dose corticosteroid therapy was defined as daily dose of more than 250 mg hydrocortisone or equivalent

Forty-five (70%) of 63 eligible patients with CMV reactivation during ICU day 3–17 could be included after matching (Fig. 1). Twenty-eight patients were matched to a seropositive patient as well as a seronegative, nine to only seropositive, and eight to only seronegative, respectively. This resulted in a study population of 118 unique patients, divided into a primary comparison (that is, 37 with CMV reactivation matched to 37 CMV seropositive without reactivation) and a secondary comparison (that is, 36 with CMV reactivation matched to 36 CMV seronegative).

### Patient and disease characteristics

Patient and disease characteristics at ICU admission were comparable between matched groups and are presented in Table 1. In the patients with CMV reactivation, median peak level of CMV DNA load was 404 IU/mL (interquartile range (IQR) 214–1370). Median length of stay in the ICU until reactivation was 9 days (IQR 6–11), which was influenced by the used inclusion criterion (that is, viremia onset between day 3 and 17 in the ICU). Of the 118 unique sepsis patients included, 81 (69%) presented to the ICU with septic shock and 80 (68%) patients had ARDS during the first week of ICU admission. In the primary comparison, the median ICU length of stay was 16 days (IQR 10–21) for patients with CMV reactivation versus 14 days (IQR 11–20) for subjects without reactivation (*P* = 0.90). This was 16 days (IQR 11–21) versus 19 days (IQR 11–27) in the secondary comparison (*P* = 0.21), respectively. Hospital mortality was 57% for patients with CMV reactivation and 46% for the matched patients without reactivation in the primary comparison (*P* = 0.35). In the secondary comparison, this was 58% versus 47% (*P* = 0.35), respectively.

**Table 1:** Characteristics of intensive care unit patients with sepsis by cytomegalovirus reactivation status

Variables	Primary comparison^a^					Secondary comparison^b^				
	CMVs + (*n* = 37)		CMV+^c^ (*n* = 37)		*P* value	CMVs− (*n* = 36)		CMV+^c^ (*n* = 36)		*P* value
Demographics and comorbidities										
Age, years	69	(63–76)	69	(62–77)	MF	68	(60–72)	69	(63–77)	MF
Sex, male	22	(60)	22	(60)	MF	23	(64)	23	(64)	MF
COPD	10	(27)	5	(14)	0.25	6	(17)	6	(17)	1.00
Congestive heart failure	4	(11)	2	(5)	0.67	1	(3)	1	(3)	1.00
Diabetes mellitus	3	(8)	9	(24)	0.11	7	(19)	8	(22)	1.00
Solid or hematologic malignancy	2	(5)	4	(11)	0.67	8	(22)	6	(17)	0.77
Chronic renal insufficiency	3	(8)	8	(22)	0.19	5	(14)	9	(25)	0.37
Charlson comorbidity index	0	(0–1)	0	(0–1)	0.29	0	(0–2)	0	(0–2)	0.81
Characteristics at ICU admission										
Hospital (A versus B)	25	(68)	25	(68)	MF	28	(78)	28	(78)	MF
Prior ICU admission	10	(27)	4	(11)	0.14	11	(31)	3	(8)	0.03
Surgical reason for admission	13	(35)	11	(30)	0.62	15	(42)	9	(25)	0.21
APACHE IV score	85	(65–103)	88	(66–114)	0.43	85	(72–96)	83	(66–111)	0.67
Septic shock	24	(65)	23	(62)	0.81	26	(72)	25	(69)	0.80
ARDS	27	(73)	26	(70)	0.80	24	(67)	23	(64)	0.80
Source of infection					0.03					0.88
- Pulmonary	20	(54)	14	(38)		16	(44)	15	(42)	
- Abdominal	5	(14)	15	(41)		11	(31)	13	(36)	
- Other	12	(32)	8	(21)		9	(25)	8	(22)	
Characteristics at t = 0^d^										
Length of ICU stay until *t* = 0 (days)	7	(6–11)	7	(6–11)	MF	9	(6–11)	9	(6–11)	MF
SOFA score	7	(4–8)	7	(4–8)	MF	7	(5–10)	8	(6–10)	MF
Corticosteroids in 4 previous days^e^	15	(41)	15	(41)	MF	17	(47)	17	(47)	MF

Abbreviations: *APACHE* Acute Physiology and Chronic Health Evaluation, *ARDS* acute respiratory distress syndrome, *CMVs+* cytomegalovirus (CMV) seropositive without reactivation, *CMV+* CMV reactivation, *CMVs*− CMV seronegative, *COPD* chronic obstructive pulmonary disease, *ICU* intensive care unit, *MF* used as matching factor, *SOFA* Sequential Organ Failure Assessment

Data are presented as medians (Q1–Q3) or frequencies (column percentages)

^a^In the primary comparison, patients with CMV reactivation were compared with matched CMV seropositive patients without reactivation

^b^In the secondary comparison, patients with CMV reactivation were compared with matched CMV seronegative patients

^c^Twenty-eight patients with CMV reactivation are included in both matched comparisons

^d^*t* = 0 is the ICU day of CMV reactivation; this length of stay also determines *t* = 0 in the matched patient without reactivation

^e^High-dose corticosteroid was defined as a daily dose of at least 250 mg hydrocortisone or equivalent

### Time course of host response biomarkers in matched groups

Baseline levels of measured host response markers were comparable between patients with and without reactivation, both at *t* = −2 (that is, 2 days prior to viremia onset) and at *t* = 0 (that is, day of reactivation onset) (Table 2). In general, this remained the case for each marker up to 10 days after CMV reactivation; the exceptions were median IL-10 levels (which were significantly higher on day 10) and median IL-6 levels (which were significantly lower on day 7) in patients with CMV reactivation compared with controls (Additional file 1: Table S1). However, these differences were not consistent across both primary and secondary comparison.

**Table 2:** Absolute levels of host response markers at baseline by cytomegalovirus reactivation status

Marker	Primary comparison^a^			Secondary comparison^b^		
	CMVs + (*n* = 37)	CMV+ (*n* = 37)^c^	*P* value	CMVs− (*n* = 36)	CMV+ (*n* = 36)^c^	*P* value
Two days before CMV reactivation onset (*t* = −2)						
IL-6	4.4 (3.9–5.4)	4.3 (3.3–4.9)	0.08	4.3 (3.7–5.2)	4.3 (3.0–4.7)	0.22
IL-8	3.6 (2.9–4.1)	3.3 (2.7–3.9)	0.35	3.5 (2.9–4.7)	3.4 (2.8–4.1)	0.23
IL-18	6.5 (5.9–6.9)	6.4 (5.9–6.6)	0.46	6.3 (5.9–6.7)	6.4 (5.9–6.7)	0.77
IP-10	6.5 (6.0–7.0)	6.3 (5.9–6.8)	0.13	6.3 (5.8–7.0)	6.3 (5.9–6.8)	0.68
N-elastase	10.6 (10.3–11.1)	10.8 (10.2–11.0)	0.96	10.7 (10.5–11.1)	10.7 (10.2–11.0)	0.42
IL-10	2.1 (1.4–3.3)	2.4 (2.0–3.1)	0.60	2.3 (1.6–3.2)	2.5 (1.9–3.1)	0.36
IL-1RA	6.4 (5.8–7.3)	6.2 (5.7–7.0)	0.55	6.4 (5.7–6.9)	6.5 (5.7–7.1)	0.63
Day of CMV reactivation onset (*t* = 0)						
IL-6	4.2 (3.1–5.0)	3.9 (3.1–4.4)	0.25	4.1 (3.6–4.9)	3.8 (3.3–4.3)	0.16
IL-8	3.5 (2.7–4.3)	3.1 (2.5–4.0)	0.36	3.6 (2.5–4.5)	3.2 (2.5–3.8)	0.16
IL-18	6.4 (5.8–6.8)	6.2 (5.8–6.8)	0.67	6.3 (5.8–6.7)	6.2 (5.8–6.8)	0.89
IP-10	6.3 (5.9–6.8)	6.4 (5.9–6.7)	0.75	6.2 (5.8–6.8)	6.5 (5.9–7.0)	0.46
N-elastase	10.3 (10.1–10.8)	10.7 (10.2–11.0)	0.11	10.7 (10.3–10.9)	10.7 (10.2–11.0)	0.85
IL-10	2.0 (1.6–3.0)	2.5 (1.9–2.9)	0.35	2.0 (1.6–2.7)	2.6 (1.9–3.0)	0.08
IL-1RA	6.4 (5.4–7.1)	6.4 (5.5–7.0)	0.81	6.3 (5.5–7.2)	6.4 (5.4–6.9)	0.54

Abbreviations: *CMVs+* cytomegalovirus (CMV) seropositive without reactivation, *CMV+* CMV reactivation, *CMVs*− CMV seronegative, *IL* interleukin, *IP-10* interferon-gamma induced protein-10, *N* neutrophilic, *RA* receptor antagonist. Biomarker levels (in picograms per milliliter) were natural log-transformed and presented as median (Q1–Q3). Measurements were performed 2 days before CMV viremia onset and at the day of onset (defines *t =* 0 in viremia patient and corresponding ICU length of stay until that day determines *t* = 0 in matched patient without reactivation)

^a^In the primary comparison, patients with CMV reactivation were compared with matched CMV seropositive patients without reactivation

^b^In the secondary comparison, patients with CMV reactivation were compared with matched CMV seronegative patients

^c^Twenty-eight patients with CMV reactivation are included in both matched comparisons

**Fig. 2:**
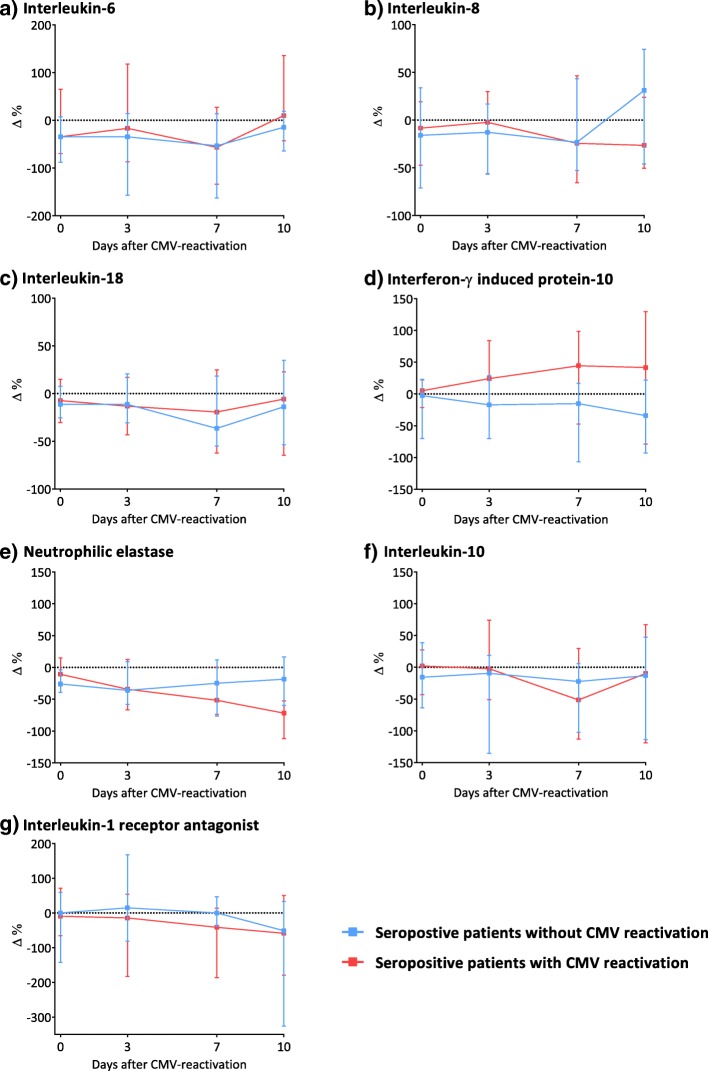
Symmetric percentage differences for time trends of host response biomarkers by cytomegalovirus (CMV) reactivation status (primary comparison). Levels (in picograms per milliliter) of pro-inflammatory (**a–e**) and anti-inflammatory biomarkers (**f–g**) were natural log-transformed, and symmetric percentage differences were calculated; ∆ % = (ln (value follow-up measurement) – ln(value at *t* = −2))*100. This measure reflects the relative difference from baseline up to the time point of interest. Results are presented as median percentage difference and Q1–Q3. Loss to follow-up (death or intensive care unit (ICU) discharge) decreased the sample size with a minimum of 11 patients per group after 10 days

Time trends of various markers within patients were described by symmetric percentage differences relative to their levels 2 days prior to CMV viremia onset (Fig. 2 for primary comparison, Additional file 1: Figure S1 for secondary comparison). For IP-10 and IL-1RA, differences in time trends were observed between patients with and without reactivation in both comparisons. Patients with CMV reactivation had a more pronounced increase of IP-10 (median percentage difference of 44% versus −15%) and decrease of IL-1RA (median percentage difference of −41% versus 0%) on day 7 after viremia onset compared with CMV seropositive patients without reactivation. For the secondary comparison, with CMV seronegative patients, similar differences in trends were observed for IP-10 (+37% versus +4%) and IL-1RA (−49% versus +10%), respectively. Of importance, sample size decreased over time because of death or ICU discharge with a minimum of 11 per patient group after 10 days (Additional file 1 Table S1).

### Independent effect of CMV reactivation on the time course of four host response biomarkers

In the multivariable mixed model analyses, CMV reactivation did not significantly affect the baseline levels of IL-6, IP-10, IL-10, and IL-1RA (Table 3). A significant decrease over time was observed in all patients for IL-6 in both the primary and secondary comparison and for IL-10 in the primary comparison only, respectively. However, CMV reactivation did not significantly affect the time trend of any of the four analyzed biomarkers.

**Table 3:** Multivariable generalized mixed model analyses relating levels and trends of four host response biomarkers to cytomegalovirus reactivation

Marker	Baseline level (*t* = −2)	Effect reactivation on level	*P* value	Time trend per day	*P* value	Effect reactivation on time trend	*P* value
Primary comparison^a^							
IL-6	5.50 (3.31–7.68)	−0.58 (−1.27–0.10)	0.03	−0.23 (−0.38 – −0.08) ^c^	< 0.001	0.07 (−0.02–0.16)	0.06
IP-10	7.23 (5.71–8.72)	−0.31 (−0.80–0.18)	0.10	−0.03 (−0.09–0.03)	0.18	0.07 (−0.01–0.16)	0.03
IL-10	2.44 (0.33–4.45)	−0.02 (−0.78–0.74)	0.94	−0.09 (−0.17 – −0.03)	0.007	0.07 (−0.02–0.18)	0.10
IL-1RA	6.12 (3.77–8.47)	−0.17 (−0.93–0.59)	0.57	−0.08 (−0.16–0.00)	0.01	0.02 (−0.09–0.14)	0.62
Secondary comparison^b^							
IL-6	4.83 (2.53–7.15)	−0.34 (−1.02–0.34)	0.21	−0.15 (−0.27 – −0.02) ^c^	0.003	0.01 (−0.07–0.08)	0.82
IP-10	6.00 (4.54–7.44)	0.14 (−0.34–0.62)	0.45	0.02 (−0.04–0.07)	0.45	0.03 (−0.06–0.12)	0.36
IL-10	1.22 (−0.60–3.08)	0.17 (−0.38–0.72)	0.43	−0.04 (−0.11–0.01)	0.06	0.02 (−0.07–0.12)	0.50
IL-1RA	4.97 (2.31–7.64)	0.06 (−0.76–0.89)	0.85	0.00 (−0.08–0.09)	0.91	−0.07 (−0.19–0.05)	0.15

Abbreviations: *CMV* cytomegalovirus, *IL* interleukin, *IP-10* interferon-gamma induced protein-10, *RA* receptor antagonist

Data are presented as coefficient of the model (99% confidence interval) derived from generalized mixed model analyses. Age and Sequential Organ Failure Assessment (SOFA) score at *t* = 0 (day of viremia onset) were included as confounders in each model because a range was used as matching criteria for these co-variables. Baseline levels were derived from intercept estimates, and the effect of reactivation on the time trend was assessed by an interaction term between time and CMV reactivation

^a^In the primary comparison, patients with CMV reactivation were compared with matched CMV seropositive patients without reactivation

^b^In the secondary comparison, patients with CMV reactivation were compared with matched CMV seronegative patients

^c^Final models for IL-6 included a polynomial time effect; the estimates for *t*^2^ were 0.01 (0.00–0.03) in the primary comparison and 0.01 (0.00–0.03) in the secondary comparison

## Discussion

We performed an explorative study to compare time trends of host response biomarkers in patients with reactivation that were matched to non-reactivating control patients who were either seropositive or seronegative for CMV. Although we initially observed differential trends of IL-1RA and IP-10 in the crude analysis, these differences did not remain in the linear mixed model analysis with adjustment for repeated measurement, loss to follow-up, and confounding. Thus, no overall and independent effect of CMV reactivation on the temporal trends of host response biomarkers following onset of viremia in patients with sepsis could be demonstrated.

The hypothesis of an immune-modulating effect of CMV is based on the observation of increased mortality and morbidity in patients with viremia without organ manifestation of CMV disease [13, 19]. Proposed mechanisms of such indirect pathogenicity are autoantibody production, enhanced inflammation, vascular damage, and CMV-induced immunosuppression [17]. Based on this hypothesis and an observed association between plasma markers and mortality in patients with ARDS [26], IL-6 was used as a surrogate endpoint in a recent randomized controlled trial that evaluated the safety of preventive antiviral treatment in ICU patients [16]. Our finding that CMV reactivation is not associated with modified IL-6 dynamics questions the suitability of IL-6 as an endpoint in clinical trials evaluating preventive therapy for CMV reactivation in ICU patients. Furthermore, time trends of other immunological biomarkers were not robustly affected by CMV reactivation.

Our study has several strengths. First, to our knowledge, this is the first study with serial measurements of the immune response following (instead of prior to) CMV reactivation. Second, our study design included two matched control groups. Because of the used matched cohort design, we could include only 45 out of 63 patients with CMV reactivation but this loss was compensated by the ability to include controls that were more comparable to those patients. Sepsis patients in the ICU are known to be very heterogeneous [27, 28]; thus, the matching reduced in theory both confounding and unwanted variation by extraneous factors. Third, by using mixed model analyses, we accounted for correlation of measurements performed within one patient by the use of random effects, which increased the statistical power to identify differences between patient groups. Moreover, this type of analysis takes into account the considerable loss to follow-up of patients and allowed us to estimate an average trend over time based on available data.

Our study also has some limitations. First, this was an explorative study evaluating multiple host response biomarkers. We chose a lower *P* value threshold of significance in order to decrease the risk of spurious findings due to multiple testing, but false-negative findings remain an accessory risk to keep in mind also when considering our study sample size. Unfortunately, a formal sample size calculation for this kind of statistical analysis was not possible. Nevertheless, we postulate that possible immunomodulating effects of CMV reactivation seem at most to be rather limited in these patients because no large differences in biomarker levels between matched groups were observed. Second, we analyzed host response biomarkers as standalone markers, which is probably a simplification of the complex immune response. However, large sample sizes are required to perform more advanced network analyses, and the integration of time series in such analyses, to our knowledge, has not been conducted before. We also measured only the plasma concentrations. Since CMV pneumonitis could be an important mediator of the pathological effect of CMV reactivation in critically ill patients, bronchoalveolar lavage samples may be additionally informative but were not available [16, 29]. Finally, we did not evaluate all potentially relevant biomarkers for CMV reactivation; thus, future studies are needed before an immunomodulating effect of CMV can be ruled out with certainty as an important pathological mechanism in previously immunocompetent ICU patients.

## Conclusions

This study could not demonstrate an independent immunomodulating effect of CMV reactivation in patients with sepsis. This finding does not lend support for the use of immunological markers as surrogate endpoints for clinical outcome in interventional studies of prophylactic or pre-emptive CMV therapy in ICU patients.

## Additional file

Additional file 1: **Table S1.** Absolute levels of host response markers during follow-up by cytomegalovirus (CMV) reactivation status. **Figure S1.** Symmetric percentage differences for time trends of host response biomarkers in patients by cytomegalovirus (CMV) reactivation status (secondary comparison). (PDF 649 kb)

## Abbreviations

ARDS: Acute respiratory distress syndrome
CMV: Cytomegalovirus
HPE: High Performance Elisa
ICU: Intensive care unit
IFN-γ: Interferon-gamma
IL: Interleukin
IP-10: Interferon-gamma–induced protein-10
IQR: Interquartile range
IU: International units
RA: Receptor antagonist
SOFA: Sequential Organ Failure Assessment
TNF: Tumor necrosis factor
TRAIL: Tumor necrosis factor-related apoptosis-inducing ligand

## References

[1] Frantzeskaki FG, Karampi ES, Kottaridi C, Alepaki M, Routsi C, Tzanela M (2015). Cytomegalovirus reactivation in a general, nonimmunosuppressed intensive care unit population: incidence, risk factors, associations with organ dysfunction, and inflammatory biomarkers. J Crit Care.

[2] Heininger A, Haeberle H, Fischer I, Beck R, Riessen R, Rohde F (2011). Cytomegalovirus reactivation and associated outcome of critically ill patients with severe sepsis. Crit Care.

[3] Limaye AP, Kirby KA, Rubenfeld GD, Leisenring WM, Bulger EM, Neff MJ (2008). Cytomegalovirus reactivation in critically ill immunocompetent patients. JAMA.

[4] Kalil AC, Florescu DF (2009). Prevalence and mortality associated with cytomegalovirus infection in nonimmunosuppressed patients in the intensive care unit. Crit Care Med.

[5] Lachance P, Chen J, Featherstone R, Sligl WI (2017). Association Between Cytomegalovirus Reactivation and Clinical Outcomes in Immunocompetent Critically Ill Patients: A Systematic Review and Meta-Analysis. Open Forum Infect Dis.

[6] Osawa R, Singh N (2009). Cytomegalovirus infection in critically ill patients: a systematic review. Crit Care.

[7] Ong DS, Spitoni C, Klein Klouwenberg PM, Verduyn Lunel FM, Frencken JF, Schultz MJ (2016). Cytomegalovirus reactivation and mortality in patients with acute respiratory distress syndrome. Intensive Care Med.

[8] Ong DSY, Bonten MJM, Spitoni C, Verduyn Lunel FM, Frencken JF, Horn J (2017). Epidemiology of Multiple Herpes Viremia in Previously Immunocompetent Patients With Septic Shock. Clin Infect Dis.

[9] Chiche L, Forel JM, Roch A, Guervilly C, Pauly V, Allardet-Servent J (2009). Active cytomegalovirus infection is common in mechanically ventilated medical intensive care unit patients. Crit Care Med.

[10] Chiche L, Forel JM, Thomas G, Farnarier C, Cognet C, Guervilly C (2012). Interferon-gamma production by natural killer cells and cytomegalovirus in critically ill patients. Crit Care Med.

[11] Clari MA, Aguilar G, Benet I, Belda J, Gimenez E, Bravo D (2013). Evaluation of cytomegalovirus (CMV)-specific T-cell immunity for the assessment of the risk of active CMV infection in non-immunosuppressed surgical and trauma intensive care unit patients. J Med Virol.

[12] Heininger A, Jahn G, Engel C, Notheisen T, Unertl K, Hamprecht K (2001). Human cytomegalovirus infections in nonimmunosuppressed critically ill patients. Crit Care Med.

[13] Papazian L, Hraiech S, Lehingue S, Roch A, Chiche L, Wiramus S, Forel JM (2016). Cytomegalovirus reactivation in ICU patients. Intensive Care Med.

[14] Gandhi MK, Khanna R (2004). Human cytomegalovirus: clinical aspects, immune regulation, and emerging treatments. Lancet Infect Dis.

[15] Papazian L, Doddoli C, Chetaille B, Gernez Y, Thirion X, Roch A (2007). A contributive result of open-lung biopsy improves survival in acute respiratory distress syndrome patients. Crit Care Med.

[16] Limaye AP, Boeckh M (2010). CMV in critically ill patients: pathogen or bystander?. Rev Med Virol.

[17] Varani S, Landini MP (2011). Cytomegalovirus-induced immunopathology and its clinical consequences. Herpesviridae.

[18] Walton AH, Muenzer JT, Rasche D, Boomer JS, Sato B, Brownstein BH (2014). Reactivation of multiple viruses in patients with sepsis. PLoS One.

[19] Freeman RB (2009). The ‘indirect’ effects of cytomegalovirus infection. Am J Transplant.

[20] Chilet M, Aguilar G, Benet I, Belda J, Tormo N, Carbonell JA (2010). Virological and immunological features of active cytomegalovirus infection in nonimmunosuppressed patients in a surgical and trauma intensive care unit. J Med Virol.

[21] Limaye AP, Stapleton RD, Peng L, Gunn SR, Kimball LE, Hyzy R (2017). Effect of Ganciclovir on IL-6 Levels Among Cytomegalovirus-Seropositive Adults With Critical Illness: A Randomized Clinical Trial. JAMA.

[22] Keustermans GC, Hoeks SB, Meerding JM, Prakken BJ, de Jager W (2013). Cytokine assays: an assessment of the preparation and treatment of blood and tissue samples. Methods.

[23] Scholman RC, Giovannone B, Hiddingh S, Meerding JM, Malvar Fernandez B, van Dijk MEA (2018). Effect of anticoagulants on 162 circulating immune related proteins in healthy subjects. Cytokine.

[24] Cole TJ, Altman DG (2017). Statistics Notes: Percentage differences, symmetry, and natural logarithms. BMJ.

[25] Cnaan A, Laird NM, Slasor P (1997). Using the general linear mixed model to analyse unbalanced repeated measures and longitudinal data. Stat Med.

[26] Parsons PE, Eisner MD, Thompson BT, Matthay MA, Ancukiewicz M, Bernard GR (2005). Lower tidal volume ventilation and plasma cytokine markers of inflammation in patients with acute lung injury. Crit Care Med.

[27] Marshall JC (2014). Why have clinical trials in sepsis failed?. Trends Mol Med.

[28] de Grooth HJ, Postema J, Loer SA, Parienti JJ, Oudemans-van Straaten HM, Girbes AR (2018). Unexplained mortality differences between septic shock trials: a systematic analysis of population characteristics and control-group mortality rates. Intensive Care Med.

[29] Blanquer J, Chilet M, Benet I, Aguilar G, Munoz-Cobo B, Tellez A (2011). Immunological insights into the pathogenesis of active CMV infection in non-immunosuppressed critically ill patients. J Med Virol.

