# Supplementary material for: Effect of cytomegalovirus reactivation on the time course of systemic host response biomarkers in previously immunocompetent critically ill patients with sepsis: a matched cohort study
Source: Crit Care. 2018 Dec 18;22:348. doi: 10.1186/s13054-018-2261-0 (PMC6299562; doi:10.1186/s13054-018-2261-0)
Supplement: Supplementary file 1 — Table S1. Absolute levels of host response markers during follow-up by cytomegalovirus (CMV) reactivation status. Figure S1. Symmetric percentage differences for time trends of host response biomarkers in patients by cytomegalovirus (CMV) reactivation status (secondary comparison). (PDF 649 kb) [file 13054_2018_2261_MOESM1_ESM.pdf]

SUPPLEMENTAL DIGITAL CONTENT

Effect of cytomegalovirus reactivation on the time course of systemic host response biomarkers in previously immunocompetent critically ill patients with sepsis : a matched cohort study

Kirsten van de Groep[1,2], Stefan Nierkens[3], Olaf L. Cremer[2], Linda M. Peelen[1,2], Peter M.C. Klein Klouwenberg[7], Marcus J. Schultz[4], C. Erik Hack[3], Tom van der Poll[5,6], Marc J.M. Bonten[7], David S.Y. Ong[1,8], *on behalf of the MARS consortium.*

Content:

Appendix I. Table S1. Absolute levels of host response markers during follow up by CMV reactivation status

Appendix II. Figure S1. Symmetric percentage differences for time trends of host response biomarkers in patients by CMV reactivation status (secondary comparison)

APPENDIX I.

Table S1. Absolute levels of host response markers during follow up by CMV reactivation status

	Primary Comparison			Secondary Comparison		
**Cytokine**	**CMVs+**	**CMV+** [a]	***p*-value**	**CMVs-**	**CMV+** [a]	***p*-value**
**Three days after CMV reactivation (*t*=3)** [b]						
	n=26	*n*=26		*n*=29	*n*=23	
IL-6	3.6 (3.1-4.5)	3.8 (3.1-4.9)	0.86	4.2 (3.6-4.7)	3.6 (2.7-4.2)	0.12
IL-8	3.1 (2.6-3.7)	3.3 (2.4-4.3)	0.69	3.3 (2.8-4.6)	3.3 (2.3-4.3)	0.35
IL-18	6.1 (5.7-6.7)	6.1 (5.8-6.8)	0.85	6.1 (5.8-6.5)	6.1 (5.8-6.7)	0.92
IP-10	6.5 (5.9-6.9)	6.5 (6.0-6.9)	0.63	6.3 (6.0-6.9)	6.6 (6.0-7.4)	0.22
N-elastase	10.3 (10.1-10.9)	10.4 (10.1-10.9)	0.56	10.5 (10.2-10.8)	10.4 (10.0-11.0)	0.73
IL-10	2.1 (1.1-2.4)	2.6 (1.7-3.0)	0.06	2.2 (1.6-2.6)	2.6 (1.6-3.0)	0.15
IL-1RA	6.4 (5.8-6.8)	6.5 (5.9-6.9)	0.87	6.3 (5.8-7.0)	6.6 (5.9-6.9)	0.96
**Seven days after CMV reactivation (*t*=7)**						
	n=14	*n*=15		*n*=19	*n*=13	
IL-6	3.9 (3.2-4.4)	3.8 (3.0-4.4)	0.66	4.1 (3.7-5.1)	3.4 (3.0-4.0)	0.005
IL-8	3.4 (2.6-3.5)	3.5 (2.7-4.1)	0.28	3.8 (3.0-5.1)	3.5 (2.7-4.0)	0.26
IL-18	6.0 (5.6-6.4)	6.3 (5.9-6.8)	0.29	6.3 (5.9-6.7)	6.3 (5.9-6.8)	0.95
IP-10	6.2 (5.9-6.6)	6.4 (6.2-6.8)	0.32	6.6 (5.8-7.2)	6.6 (6.3-6.9)	0.55
N-elastase	10.2 (9.8-10.7)	10.5 (10.4-10.7)	0.19	10.3 (9.9-10.9)	10.6 (10.4-11.0)	0.39
IL-10	1.7 (0.7-1.8)	2.3 (1.8-2.7)	0.03	2.0 (1.4-2.6)	2.3 (1.9-2.7)	0.45
IL-1RA	6.2 (3.7-6.4)	6.2 (4.7-6.7)	0.84	6.5 (6.0-7.1)	5.9 (3.1-6.4)	0.07
**Ten days after CMV reactivation (*t*=10)**						
	n=11	*n*=11		*n*=15	*n*=11	
IL-6	3.7 (3.1-5.0)	4.7 (3.4-5.3)	0.22	4.4 (3.6-4.8)	4.2 (2.9-4.7)	0.36
IL-8	3.2 (2.5-3.8)	3.6 (2.0-4.4)	0.87	3.8 (2.8-4.8)	3.6 (2.0-4.4)	0.31
IL-18	6.1 (5.0-6.5)	6.4 (6.0-6.6)	0.31	6.4 (6.2-6.7)	6.2 (5.9-6.6)	0.55
IP-10	6.2 (5.8-6.9)	6.8 (6.1-7.2)	0.34	6.3 (5.7-7.3)	7.1 (6.3-7.4)	0.24
N-elastase	10.4 (9.8-10.5)	10.4 (9.8-11.0)	0.49	10.5 (9.9-10.8)	10.6 (9.8-11.1)	0.70
IL-10	1.6 (1.0-2.0)	2.7 (1.8-3.1)	0.002	1.8 (1.5-3.1)	2.7 (1.7-3.1)	0.45
IL-1RA	3.1 (3.1-6.2)	6.2 (5.8-6.7)	0.19	6.2 (5.1-6.9)	5.8 (3.1-6.3)	0.48

CMVs+: cytomegalovirus (CMV) seropositive without reactivation. *CMV+:* CMV reactivation. *CMVs-*: CMV seronegative. *IL:* interleukin. *IP*=interferon-γ induced protein. *N*: neutrophilic. *RA:* receptor antagonist. Biomarker levels (pg/mL) were natural log transformed and presented as median (Q1-Q3). Due to ICU discharge or death sample size decreased over time to a minimum of 11 patients per group after ten days.

[a] 28 patients with CMV reactivation are included in both matched comparisons.
[b] *t*=0 is the ICU day of CMV reactivation, this length of stay also determines *t*=0 in the matched patient without reactivation.

APPENDIX II.

Figure S1. Symmetric percentage differences for time trends of host response biomarkers in patients by CMV reactivation status (secondary comparison)

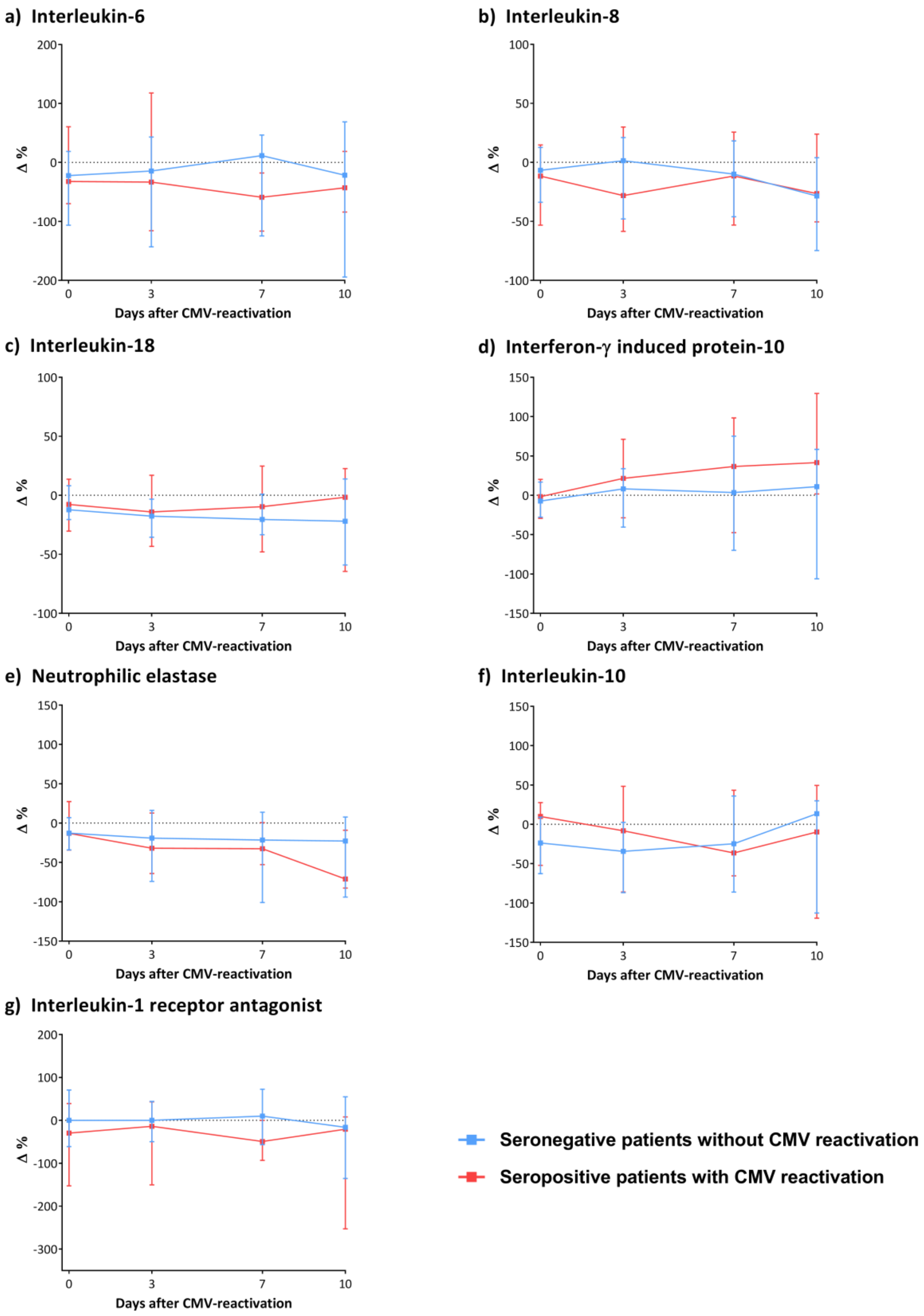

Levels (pg/mL) of pro-inflammatory (figure *a-e*) and anti-inflammatory biomarkers (figure *f-g*) were natural log transformed and symmetric percentage differences were calculated; Δ %= (ln (value follow up measurement) – ln(value at *t*=-2))*100. This measure reflects the relative difference from baseline up to the time point of interest. Results are presented as median percentage difference and Q1-Q3. Loss to follow up (death or ICU discharge) decreased the sample size with a minimum of 11 patients per group after 10 days.
